# Between the genotype and the phenotype lies the microbiome: symbiosis and the making of ‘postgenomic’ knowledge

**DOI:** 10.1007/s40656-023-00599-y

**Published:** 2023-12-06

**Authors:** Cécile Fasel, Luca Chiapperino

**Affiliations:** STS Lab, Institute of Social Sciences, Faculty of Social and Political Sciences, University of Lausanne, 1015 Lausanne, Switzerland

**Keywords:** Microbiome, Postgenomics, Symbiosis, Development, Model organisms, Experimental artefacts, Drosophila

## Abstract

Emphatic claims of a “microbiome revolution” aside, the study of the gut microbiota and its role in organismal development and evolution is a central feature of so-called postgenomics; namely, a conceptual and/or practical turn in contemporary life sciences, which departs from genetic determinism and reductionism to explore holism, emergentism and complexity in biological knowledge-production. This paper analyses the making of postgenomic knowledge about developmental symbiosis in *Drosophila melanogaster* by a specific group of microbiome scientists. Drawing from both practical philosophy of science and Science and Technology Studies, the paper documents epistemological questions of artefactuality and representativeness of model organisms as they emerge in the day-to-day labour producing and being produced by the “microbiome revolution." Specifically, the paper builds on all the written and editorial exchanges involved in the troubled publication of a research paper studying the symbiotic role of the microbiota in the flies’ development. These written materials permit us to delimit the network of justifications, evidence, standards of knowledge-production, trust in the tools and research designs that make up the conditions of possibility of a postgenomic fact. More than reframing the organism as a radically novel multiplicity of reactive genomes, we conclude, doing postgenomic research on the microbiota and symbiosis means producing a story that deviates from the scripts embedded into the sociotechnical experimental systems of post-Human Genome Project life sciences.

## Introduction

Gut microbiota and corresponding microbiome^1^ have been copiously funded and increasingly prolific research objects for the past 20 years. In 2014, introducing a Gut Microbiome review series in the *Journal of Clinical Investigation*, microbiologist Martin J. Blaser bombastically wrote about “the microbiome revolution” (Blaser, 2014). He was not referring so much to scientific interest in the *microbial* communities we carry, which can be said to date back to the early hours of microbiology. Rather, he was talking about the technological, social, and epistemic transformations that led to the birth and proliferation of *microbiomics*. On the one hand, Blaser writes, “new technologies including high-throughput DNA sequencing and bioinformatics exposed our microscopic interior in ways analogous to the roentgenograms of the prior century” (p. 1462). Much like X-rays—or, for that matter, neuroimaging at the dawn of the twenty-first century (Rose & Abi-Rached, 2013)—the availability of genomic technologies has made visible our symbiotic and microbiotic selves in ways unavailable to the gaze of past researchers. The genomic diversity and heterogeneity of the human body; the symbionts’ relevance for human biological functions; the effects of changes in symbiont composition have all become objects of inquiry reframed by the availability of genomic technologies. On the other hand, Blaser underscores that metagenomics developed in a context in which genomic investigations were being "infused with concepts from ecology, molecular taxonomy, and evolutionary medicine”: a conceptual move that produced a renewed relevance of those “systems approaches, [that were] once intractable and also anathema to reductionist experimentation” (Blaser, 2014, p. 4162). Thus, Blaser calls “the microbiome revolution” something that lies beyond a major technological breakthrough. This alleged revolution is the demise of what he calls an “Ancient Regime”: that of an “overwhelming emphasis on the human cell, genes, and genome” (ibid). The rise of microbiome science, he argues, entails the abandonment of dominant host- and gene-centric explanations for biological phenomena (including health and disease), thus giving way to a different moment in the history of biology, characterized by attention towards symbiosis, holism and emergentism mainly illustrated by a widespread conception of the body as an ecosystem (Dupré, 2010).

Emphatic claims of “microbiome revolution” aside, Blaser is not alone in noticing that microbes burst into biomedical research in ways that contribute to a moment of “transition and contestation” in the life sciences (Richardson & Stevens, 2015a, p. 46). Several scholars interested in the scrutiny of post-Human Genome Project (HGP) research (such as epigenomics, metabolomics, transcriptomics, etc.) have called this moment “postgenomics”. Yet, the term is itself polysemic and contested (Griffiths & Stotz, 2013; Link, 2018; Morange, 2018, 2006). On the one hand, postgenomics is presented as a shift and a practical turn in the life sciences, which departs from genetic determinism and reductionism (i.e. Blaser’s alleged Ancient Regime) to embrace holism, emergentism and complexity. According to Dupré (2010), metagenomics and microbiomics epitomize this postgenomic view of the organism for the following reason: not only do they show that the nuclear genome is reactive in determining phenotypes (including health and disease), but rather that the organism is made up of a multiplicity of reactive genomes. Plasticity, openness to the environment, variation and stochasticity are the defining characteristics of organisms as symbionts rather than simply genotypes transitioning into phenotypes.

On the other hand, others retort these claims may be sheer rhetoric since they are neither new, nor they are specific to this moment in the historiography of biology (Wade, 2016). Plasticity and organicist thinking in biology should be treated with much more nuance: a) historically, they certainly predate the aftermath of the HGP, if not modern biological sciences (Chiapperino & Panese, 2019; Meloni, 2019); epistemologically, they constitute approaches that have co-existed with(in) molecular biology even under Blaser’s so-called Ancient Regime (Gilbert, Sapp, & Tauber, 2012; Peterson, 2017; Pigliucci, 2001). If anything, Morange argues (2018), postgenomics should be used as a label for the post-HGP tools used by biologists (i.e. advanced sequencing and multi-omic technologies). Under this reading, postgenomics should designate the centrality of the sociotechnical infrastructures of post-HGP research (O’Malley & Dupré, 2005), and not the access to a hitherto unseen holistic logic of life in biology (MacLeod, 2015).

These epistemological tenets of postgenomics have attracted extensive scrutiny (Griffiths & Stotz, 2013, Ch. 5; Morange 2006; Meloni, 2016; Richardson & Stevens, 2015). As nicely summarized by Talia Dan-Cohen, these assessments cluster into a “glass-half-full” versus “glass-half-empty” logic (Dan-Cohen, 2016, p. 908). Fewer have instead studied the specific “facts and processes” that, within the contemporary laboratory, enable scientists to walk the tightrope “at the fuzzy boundary *between* the trivial and the complex” in their practice (Rheinberger, 1997b: S247, original emphasis). Taking a philosophy of science-in-practice approach (Ankeny et al., 2011; Boumans & Leonelli, 2013), this paper explores these theoretical, methodological, and contextual processes in the field of microbiomics. The paper follows a specific group of microbiome researchers working on developmental symbiosis in *Drosophila melanogaster.* It documents a shift in these scientists’ understanding of development from a process explained by linear genotype-to-phenotype (G to P) transitions, to one encompassing symbiosis and modulation by environmental factors (specifically, under-nutrition). The article probes this shift as emergence of a postgenomic fact about development in the two senses laid out above: primarily, as understanding of development characterized by plasticity, openness to the environment and metagenomic interactions, but, more importantly, as knowledge resulting from a specific assemblage of experimental systems (Rheinberger, 1997b). Specifically, we detail how a symbiotic description of development in the lab emerges at the crossroad of the unexpected behavior of the lab’s model organism in experimentation and the tinkering with the group’s established genomic experimental systems. In fact, when facing a stalemate in the identification of the genetic elements associated with, or causally relevant to, a developmental phenotype, these scientists retuned their experimental instruments into a different objective. This was studying the fly’s development as a property of symbiotic interactions—that is, not just as the (host) genotype transitioning into phenotype.

More than the success of this endeavor, however, the paper analyzes the struggle and resistances faced by these scientists in the publication of their results.Their experimental approach to development faced in fact strong opposition in peer reviewing, leading to publication only upon its seventh round of submission. Below, we take as materials all the written and editorial exchanges involved in this troubled publication. These written materials are part and parcel of the system of knowledge production (Rheinberger, 2003) and gave us privileged access to the theoretical, methodological, and interpretative dispute raised by the group’s work. Chiefly, we qualify this dispute as the group having produced a use of their experimental systems going outside their usual “script” (Akrich, 1997). As argued by Akrich (1997), the scientist or innovator’s work amounts also to inscribing a script, a vision of or hypotheses about the world and the actors (relationships, causality, morality, circulation, economies, hierarchies, etc.) into technical and scientific objects. In the case of model organisms like *Drosophila*, this script consists of established hypotheses and methods, materials, resources and infrastructures to conduct research in this field. As Hui, the postdoc and first author of the study we follow here summarizes in an interview, their article “went through lots of rebuttals”, but “nobody asked for an additional experiment”. The controversy was “just [in] the idea, *the idea itself*” that resulted from the researchers’ tinkering with the scripts defining the tools of their lab (emphasis added). To further characterize the epistemological tenets of this dispute we also report on the questions of artefactuality and representativeness of the model raised in these exchanges.

Our close examination of the pragmatic day-to-day labour feeding into rhetorics of postgenomics or the “microbiome revolution” restitutes a different reading of this “moment of “transition and contestation” in the history of the life sciences (Richardson & Stevens, 2015, p. 46). First, we argue for resisting the idea that microbiome research *either fails or succeeds in enacting a paradigm-shift* into postgenomics. Specifically, we maintain that peer exigencies, standards of knowledge-making, socio-technical infrastructures and tools (including model organisms)—more than theoretical disputes and changes—make questions of symbiosis pertinent and doable within post-HGP research. Our case study contributes therefore an empirical take on those philosophical analyses underlining how postgenomic science is neither the assemblage of a novel holistic ontology of the organism, nor simply the wolf of genetic reductionism and determinism in sheep’s clothing (Guttinger & Dupré, 2016). Rather, while it is enacting a trend, a latent injunction about the way post-HGP research should be conducted, it is also heavily constrained by epistemic (material, technological, experimental) and social (peer expectations) conditions of possibility of the ensuing facts. Second, our paper offers original documentation of the struggles attached to the alignment of post-HGP experimental systems into a postgenomic agenda of research. This innovation entails *taking considerable risks* for the average microbiomics laboratory. There exists, in other words, a discrepancy between established ways of knowledge-making, tools and epistemic practices of today’s molecular biology labs, and the drive to see the body and its phenotypes as a plastic assembly of human and microbial cells and genomes. If anything, we conclude, postgenomics is an intricate and fraught relation among ideas, tools, and approaches of post-HGP life sciences, which is far from coherently marking a definite moment in the historiography of biology.

## Social studies of microbiomics

The same year Blaser proclaimed the advent of the (postgenomic) “microbiome revolution” and the demise of the Ancient Régime, anthropologists Paxson and Helmreich wrote about the significance of the “microbial turn in recent biology” (Paxson & Helmreich, 2014). In their view, this was symptomatic of a shift in the representation of nature—one that turned away from an “age of biological control” where microbial abundance was conceived as perils; one that staged instead a nature that is underdetermined, full of promises and futures, where microbial life becomes a model ecosystem—a model of how entangled, relational and dynamic life “could, should, or might be” (p. 168) at every scales. The two anthropologists, as well as other scholars, describe how this “turn”—be it called microbial or probiotic (Lorimer, 2020)—produces a new characterization of micro- and macrobial bodies that would be ontologically relational and ecosystemic. A gesture that has also revived long-standing philosophical debates on the contours of biological individuality and the definition of organism (see for example Baedke, 2019, 2019a, b; Pradeu 2016). Crucially, other scholars have tamed the illusion of rupture that such narratives may produce, pointing to the need of a higher attentiveness to the longue durée of these ideas (Sangodeyi, 2014; Brives & Zimmer, 2021; de Guglielmo, 2021; Anderson, 2004). Other scholars have raised concerns about unexamined political undertones of microbiome research, such as the naturalization of racial, ethnic, or gendered categories in these research practices (Benezra, 2020; Helmreich, 2014), or the big-datafication of undernutrition these further, as well as the incommensurability between multi-omic approaches to health and the thickness of day-to-day experiences of illness (Benezra, 2016). Concerns have also been raised about the destructive impact of certain types of microbiome research and their paradoxical reinforcement of the illusion of separation of bodies from their milieu (Zimmer, 2019). Some scholars, therefore, point towards the need of novel interdisciplinary alliances reflecting upon the social dimension of microbiome research. These amount to (i) the incorporation of justice concerns in the debate about microbial diversity on a global scale (Ishaq et al., 2021); or, (ii) the development of an agenda for social science research on the human microbiome that fosters citizen participation (Greenhough et al., 2020). Such calls have been discussed in terms of interdisciplinarity (Greenhough et al., 2018), participatory (Lorimer et al., 2019) and collaborative (Mäkinen, 2018) research settings. Still rare are the documentations of the epistemic/epistemological dimensions of this postgenomic field. The proliferation of microbiome-related research has been discussed in theoretical terms due to the merging and coexistence of different taxonomic systems (Sommerlund, 2006), multispecies research and the rethinking of immunity (Lorimer, 2019; Lorimer et al., 2019), challenges to causality (Gontier, 2021; Lynch, Parke, & O’Malley 2019) and multi-level research approaches to complex biological systems (O’Malley et al., 2014). Yet very few studies have documented the technoscientific settings (e.g. research designs, tools, ideas, relations, collaborations, etc.) that make up the so-called microbial turn. What do distinct attempts to enact coherently such would-be ontologically relational and ecosystemic bodies look like in practice? This is the question that we explore below.

## An experimental system and its context

In what follows we document a story pivoting around symbiosis, microbial cells, and genomes. Our materials are drawn from (what we call) the *Integrative Physiology of Symbioses Lab (IPS Lab)* located at the Pluridisciplinary Institute of Functional and Integrative Genomics (PFIG).^2^ The lab was founded by Hal, a European Research Council grantee and a geneticist specialized in the use and study of *Drosophila*—a “Drosophilist” as he self-identifies. We analyze one line of research of the IPS Lab, which involves mainly Hal and Hui, a postdoc cell-biologist and ‘Drosophilist’ trained in Stanford, USA. Yet, the group needs a variety of backgrounds to pursue its objectives: some members are experts on *Drosophila*, others are bacteria specialists with a track record on the microbes employed in the lab. They are geneticists, bacteriologists, and microbiologists, but also cellular biologists. Aside from a heterogeneous team of researchers, Hal’s lab includes a heterogeneous network of technical experts, spaces, rooms, tubes, temperatures, and instruments that can produce the “*Symbiosium* Growth Effect” (see below) and make it a collection of manipulable and measurable phenomena.

We study the practices of the IPS Lab from the entry point of all the written and editorial materials involved in the troubled publication of a research paper where Hui is the first and main author (while Hal the last coordinating one). These materials include written exchanges between the team, editors, and reviewers, as well as the 6 versions of the original manuscript that underwent both minor and major modifications across 3 rounds of peer-review and 6 editorial rejections in 7 journals. The publication process, between the submission to Journal 1 and the acceptance by Journal 7 lasted 14 and a half months. As a complement to these documents, we also draw from 13 interviews with scientists working in microbiome research to provide context to the IPS Lab’s research. We also interviewed Hal and Hui on 5 occasions and accompanied the analysis of their experimental system dissecting the Symbiosium-Growth Effect with a series of lab observations.

### The fly

*Drosophila melanogaster* is well known as a model organism and as a major catalyzer of the molecularization of developmental biology (Kohler, 1994; Morange, 2020). Hal’s lab uses *Drosophila* to produce knowledge about host–symbionts interactions; a pattern of use of this model organism that is recent but still intimately linked to *Drosophila*’s career in genetics and molecular biology (Ma et al., 2015). A seminal paper making this use of *Drosophila* offers the main reason to choose this organism also for the manufacturing of symbiont knowledge: “the enormous diversity of the resident microbiota community of the mammalian gut [...] and the genetic complexity of the host [...] make it difficult to clearly establish the molecular links that would clarify the[se] relations [...] at the organism level” (Ryu et al., 2008, p. 782). Thus, *Drosophila* is considered extremely suitable to the life sciences’ need to employ experimental devices that tame the “complexity” of so-called higher organisms, including human beings (Ankeny, 2007; Ankeny & Leonelli, 2011; Ankeny, 2000). It constitutes “a genetically amenable model organism, which harbors an extremely simple gut commensal structure” (Ryu et al., 2008, p. 782). Also, the fly can be easily manipulated with an interventional design that singles out molecular mechanisms of commensal–gut interaction. This typically entails decomposing host–microbe symbiosis and testing their interaction through gnotobiotic states (i.e., a state of an organism in which all the microorganisms living in symbiosis are either known or excluded), including axenicity (i.e. the state of being germ-free). Controlling how different symbionts assemble, as well as their genetic characteristics, allows scientists to build a mechanistic explanation of the phenotypic effects of any host–microbe interaction—i.e., a depiction of the “orchestrated functioning” of host and microbe components that are responsible for a given phenotypic outcome (Bechtel & Abrahamsen, 2005, p. 424; Bechtel 2013). For instance, several scientists have shown how different *Drosophila* genomic backgrounds impact microbial effects on phenotype, or how variations in microbiota add to the effects of this genetic variation in flies (Martino, Ma, & Leulier, 2017). Hal’s lab thus walks in the footsteps of the history of *Drosophila* as a model organism (Mohr, 2018; Keenan & Shvartsman, 2017; Kohler, 1994): it is a powerful research tool to perform functional (genetic) annotation studies of biological differences. Yet, the lab’s research also pursues a growing research program that tries to extend the generalizable, controlled, and mechanistic toolkit of *Drosophila* into a cutting-edge question of postgenomic life sciences: identifying the molecular chains of events that link the host’s genes to the symbiont’s genes and back.

### The bacterium

*Drosophila* is not the only model organism at the core of the IPS Lab’s experimental systems. There is also a bacterium: which we will call here *Symbiosium*. In an interview, Hal narrates that he founded the laboratory with the aim of “dissecting the mechanism” of the role of *Symbiosium* in promoting the fly’s growth. Hal and his colleagues had in fact noticed—and *bona fide* demonstrated to their peers—that underfed axenic flies when reinoculated with *Symbiosium*, catch up with the normal growth of flies raised with normal flora. He baptized *Symbiosium*’s action over flies’ growth the “*Symbiosium* Growth Effect”. “This result being a singular and rather original one”, Hal recalls, “I said to myself: I have a niche there; I have a great result and *there is everything to do*, on the bacterial side, on the *Droso* side. So, I applied to set up my lab” (Interview Hal). The “everything”, which Hal refers to here, leads us back to the vision of science inscribed or scripted (cf. Akrich, 1997) into the conjunction of these two experimental organisms, *Symbiosium* and *Drosophila*, in the postgenomic lab. In fact, technical objects, Akrich argued, “contain and produce a specific geography […] of causes” (Akrich, 1997, p. 207). This implies that adopting a tool means adopting specific questions that are baked into these tools, and it means also adopting the tools’ ways to answer them. In the specific configuration at issue, Hal’s words underline that this combination of tools and models yields a knowledge gap about the mechanistic effects of symbionts not only in growth and development but also in locomotion, aging, cancer development, susceptibility to infections, etc. Also, his words highlight the answers the tools of the post-HGP lab (i.e., advanced sequencing and multi-omic technologies) can offer to disentangle these interactions: whether, for instance, these are controlled by regulatory processes, genomic differences or else in both hosts and microbes. According to Hal, the *Drosophila-Symbiosium* mono-association model is “a *biological system* simplified to its extreme” (emphasis added)—the biological system simplified here being an ecological view of living beings as heterospecific and plastic assembly of host and microbial cells and genomes. Of note, the complexity of such multispecies integrations is made amenable to the linearity of a one-to-one relationship between genetically tractable and manipulable organisms that is common practice of experimentation.

## Changing research question, reorganizing the tools

In this section, we document how Hui and Hal gradually tuned their lab work into configurations that are unusual to the IPS Lab’s practices we just introduced. As we shall see, tweaking and experimenting with the instruments at their disposal—more than a theoretical interrogation of organismal complexity—led the laboratory into producing an understanding of development as regulated by complex causal relations and a heterogeneous set of biological systems including the genome, the microbiota and the environment.

### A tool comes with the questions it can ask

The project we focus on begins with the integration of a tool into the laboratory’s experimental network: the *Drosophila* Genetic Reference Panel (DGRP). Hal decided to integrate the DGRP in his lab because of shared workplace and colleagues within the community developing, discussing, using, and publishing about this nascent and “a bit trendy” tool (interview Hal). He thought that the DGRP could be fruitfully aligned toward the mechanistic deciphering of the *Symbiosium* Growth Effect.

The DGRP is “a community resource” (Mackay et al. 2012)—the community here being that of the Drosophilists, the “fly people” (interview Hui). It is a model population of ~ 200 *Drosophila* strains derived from a natural population (harvested in a flea market). Each line is a pure line, which is the homozygous result of repetitive selective inbreeding. These DGRP lines are said to “contain” in their standardized genome a sample of *natural* genetic variations. Each of the lines is fully sequenced and the raw sequencing data are available online for the whole community. Importantly, the usefulness of the tool also relies on every researcher that uses the DGRP sharing their data on a publicly available database, as well as on a website allowing the researchers to perform Genome Wide Association Study (GWAS)^3^ by simply logging properly coded phenotypic data. The DGRP is a gene-oriented tool and a pivotal element of the mechanistic toolkit of Drosophilists. Its aim is to allow the fly community to “understand the relationship between molecular genetic variation and variation in quantitative traits” (Mackay et al., 2012, p. 173). The mapping of this relationship is achieved by statistically associating molecular genetic variations with phenotypic variations, sometimes under various controlled environments. In many cases as in ours, this entails performing GWAS. The DGRP was first constituted around 2010 after a few years of intense human GWAS production and publications. The problem with human GWAS is that yielded candidate genetic variations are not accessible to any causal probing: not all statistical associations represent relevant biological activity. The genetic differences observed may in fact not be causally relevant to the phenotype under study. By contrast, in flies, genetic manipulation based on the DGRP allows scientists to create model organisms with a standardized genetic background that are mutants for any given gene (e.g. knock-out experiments). Thus, the DGRP tool is a major enabler of the research program of the IPS Lab: it articulates a controlled (and shared) genomic database with molecular mechanistic tools that allow the scientists to probe causal relationships between host, microbes and their genetic variation. For the geneticist and DGRP-project head Trudy Mackay, with the DGRP “[we] can begin to really understand *the* biology behind the association[ist]” type of knowledge yielded by GWAS (Hughes, 2010, emphasis added). As Hal puts it, we can “dissect the genotype-to-phenotype map”.

In the analytical terms of Akrich's “script”, these material, social and historical factors participate in inscribing into the DGRP tool specific “hypotheses about the entities that make up the world into which the object is to be inserted” (Akrich, 1997, pp. 207–208). In plainer terms, adopting the DGRP tool in the lab means adopting the questions that are to be asked with these tools, as well as conforming to the ways the tools can answer them. The DGRP lines came in the lab bringing with them the following question: *What are the host’s genetic drivers of the Symbiosium Growth Effect?* Thus, in the intent to perform a GWAS, the team measured the trait of interest (i.e. the growth-promoting effect of *Symbiosium* inoculation measured through length of the developing flies) in different lines to see if certain genotypes benefit more than others from the *Symbiosium* Growth Effect. This step of lengthy measuring is called phenotyping. Then the values are to be encoded in the DGRP communitarian website to perform an automated GWAS, aiming at singling out certain genes that present a strong association with growth-effect differences. Then, ideally, the lab team would intervene into the candidate genes (e.g. inactivate them), demonstrate causal relationships and end up identifying a ‘molecular mechanism’ linking—by means of a material chain of molecular and protein events—those genes to the biological action of *Symbiosium*. The aim, therefore, is to combine the statistical power of genome-wide methods (here: GWAS) with the opportunities *Drosophila* offers to produce mechanistic knowledge (here through the manipulation of candidate genes). The geography of causes (cf. Akrich, 1997) baked into this combination of experimental systems places genes and DNA sequences as the main explanatory element that produces the phenomenon. More than a deliberate choice of the lab this specific mechanistic explanation is what the experimental system reveals.

### Changing the research question, redrawing the geography of causes

Yet, as “generators of surprise” (Rheinberger, 2011, p. 314), these experimental systems also did not behave as imagined by the IPS Lab scientists. Unbeknownst to them, their scripted gene-centric program had to be reworked:

“ …we had a naive vision where we thought GWAS would allow us (…) to identify *Droso* genotypes where, perhaps, *Symbiosium* was no longer beneficial. Trying to understand the genetic basis of the effect of Symbiosium; that has always been the question. But we realized that GWAS is not going to be relevant for this. Yet, on the other hand, *we had this buffer effect*, so we did this other story.” (Hal, interview; emphasis added)

In fact, the question of *which fly genes are “responsible” for the positive effect of Symbiosium on their growth* is soon abandoned because of two problems. The first one is that the phenotyping step as it was conceived was too demanding^4^ in terms of time and “man-power” (team’s answer to a referee). Hui, who *woman*-powered this work, could not test and measure more than 53 lines. The GWAS was thus underpowered. Yet, besides practical considerations, the major problem with the study was rather that genetically heterogeneous flies *do not* show a differential benefit from their association with *Symbiosium*.^5^ The flies rather show *a differential in the variance of their growth depending on their association with Symbiosium*; in other words, axenic (i.e. germ-free) flies can be very different in size (high variance) while mono-associated flies, *regardless of their genotype*, largely cluster around the same sizes (low variance). Thus, the phenotype seems to be more homogeneous, stable, or ‘robust’ in the larvae associated with *Symbiosium* than in the germ-free (henceforth GF) ones. This observation challenges the reasoning about the mechanism baked into the lab’s tools: the genotype, it turns out, is not the causal component yielding the phenotypic differential. Within the experimental setting of the IPS lab, it’s the symbiotic relationship itself that turns into the agent that is difference- and trait-making.

Hui has previously read the work of American biologists Susan Lindquist and Susan Rutherford. The phenomenon she observes excitingly reminds her of the concept of *genetic buffering*, which those scientists introduced in 1998 (Rutherford & Lindquist, 1998). With ‘genetic buffering’ they sought to bring into molecular biology Waddington’s concept of canalization.^6^ Hui hypothesizes that *Symbiosium* might be playing the same role Lindquist and her collaborators assigned to certain proteins in development: what if, she asked, *Symbiosium* drives the attenuation of the phenotypic manifestations of genetic variations that gets unmasked by environmental stress? During an interview, she narrates: “I went back and read the [1998 Nature] paper again, and of course, thinking about buffering, then I started reading about robustness, canalization, and then I started reading about Waddington, his classic paper with *Drosophila*” (Interview Hui). Not only at this stage of the lab’s work is the study not doing very well regarding its scripted questions and answers (cf. Akrich, 1997). But, actually, the observations genuinely lead it in a whole different direction.

The team decides it is better to re-tune its instruments and preliminary data towards the exploration of their unfitting observation. What started as a study fully inscribed into the (tools for dissecting the) genetics of *Drosophila*’s growth and development in the presence/absence of *Symbiosium* gets re-oriented towards a thoroughly different question: the role of *Symbiosium* and symbiosis in phenotypic development and canalization. More specifically, the question turns into searching an extra-genetic mechanism operating the *Symbiosium* buffering effect: that is, dissecting the role of symbiotic relationships in buffering the impact of genetic variation on development in the face of an environmental stress (i.e. the flies’ undernourishment). But this task is far from manageable from the standpoint of the lab’s experimental systems. As others have recognized in publication, Hui and Hal are confronted to the dearth of evidence on these processes within their experimental setting. As argued by evolutionary biologists Mónica Medina and Joel Sachs, to dissect the “entangled banks” (Medina & Sachs, 2010, p. 129) that is a symbiont, biologist should “broaden [their] understanding of how host–symbiont interactions are actually taking place at the cellular level” (ibid, p. 135). Yet, a “whole suite of postgenomic techniques”, they laconically suggest, are probably in need of development to fulfill this task (ibid, p. 136) Thus, for lack of a straightforward way to dig deeper on their observation, the IPS Lab members craft a new outlook over their model. Primarily, the scientists re-analyze the data with the aim to ground the observation on a statistical demonstration. That means showing the *relationship* between *Symbiosium-Drosophila—*and not any of the flies’ (or *Symbiosium*’s) genetic components—produces a robust and homogenous phenotype of the flies (i.e. it prevents environmental stressors to amplify the phenotypic effects of cryptic inter-individual genetic variations). These manipulations restitute 1) that the variance of length is significatively superior in GF larvae than in the mono-associated ones; and (2) a relative uncoupling in the genotype-to-phenotype association in presence of the bacteria (i.e. the length of the flies is more determined by genotype in GF larvae than in the mono-associated ones; in the mono-associated flies, bacteria was more of a trait-maker than the genotype itself was). In plainer terms, symbiotic relationships (not genetic variants) nudge developmental processes. And this reversal of the causal geography of development in symbiosis (cf. Akrich, 1997) becomes a novel way to articulate and interrogate the lab’s experimental systems.

Secondly, the team probes *the generalizability of their evidence*. Is the buffering effect a phenomenon that occurs into the wild, or is it just an artefact of laboratory practices employing inbred lines (i.e., DGRP), GF conditions, mono-associated cultures, and major environmental stressors? The goal of the lab members is to show that “buffering is not just an esoteric DGRP phenomenon” (Team’s answer to a referee). *Symbiosium*’s buffering actions take place *regardless of* the flies’ genetic background, *but* in a heavily controlled setting in terms of genetic diversity (i.e., the DGRP inbred lines) and gnotobiosis. Thus, to show that this is not an artefact of working with pure lines, IPS Lab members decide to tweak their experimental systems. On the one hand, this translates into breeding the DGRP lines with one another, which produces a genetically heterozygous population mimicking the diversity of fruit flies into the wild. This yields data on buffering in flies with a genetic background that is no longer “pure” and controlled as in the DGRP. On the other hand, the lab reproduces the experiments/results that led to the primary observations of the buffering effect with a wild-type-like population. This means attempting to reproduce the experiment with fly lines holding wild-type microbiota. This means showing the buffering effect beyond a very simplified one mono-associated with *Symbiosium* (for this step, the team simply catches wild flies with rotten tomatoes in a garden).

## Negotiating representativeness of the unscripted uses of experimental systems

The team shows excitement about their results in both the first drafts of the manuscript and the interviews we conducted. Several passages discuss how thought-provoking their experiments were, while the informants elaborate in interviews on the implications of their study for knowledge of biological complexity, symbiotic conceptions of the living and holobionts' coevolution.

Yet, these efforts are found unsatisfactory and not worth publishing by many. Referees throughout the different submissions are very polarized, some of them being, as Hal says, “very nasty”; some being very enthusiastic. The latter are those who praise the creativity of the study and the “fascinating and surprising results” (referee 2 journal 3). The former largely dominate the conversation in number, but also in substance: their arguments convince several editors to pass on the paper. Criticisms are mostly conceptual in nature—“[it was] just the idea, *the idea itself*” says Hui in an abovementioned interview excerpt—leaving little room for further experiments that could make the paper publishable. Answering to the very first submission, the journal editor states that the “study does not provide *the type of development* that would warrant publication” (emphasis added). In their exchanges with [Journal 3], the first referee writes that “the story only takes the reader halfway to the conclusion”, severely adding that “not enough work has been done”. Certain standards in the construction of the claims, reviewers allege, have not been respected. But what is exactly lacking in the buffering study? What type of work has not been done? Which element, information or demonstration is missing? As a matter of fact, the vast majority of the editors and referees—as well as the team itself to a certain extent—agree on the fact that *a certain work* has not been done properly, or at all, and that consequently the story lacks a satisfactory development worth publishing. But not everyone agrees on *what* should have been done, done differently, or said, and how, for the story to be worth sharing.

This article is, in this sense, a form of controversy analysis. Making sense of the buffering study is a question of unfolding, in the words of Pestre, the “making and negotiation of *meaning* between actors” (Pestre, 2007). *What does the buffering study mean and what does it fail to mean?* The thing that strikes upon reading these exchanges is the utter variability of the reviewers’ interpretations of the importance of the buffering effect story; i.e. which community of scientists it is aimed at (a niche knowledge, or a knowledge that is relevant for the field of biology in general). For instance, in their rebuttal emails, editors of [Journal 1 and 2] argue respectively that “the study would find a more suitable audience” elsewhere, and specifically in “a more specialized journal”. Referee 2 from [Journal 3] says that “this finding has important evolutionary implications”, which are presumably of a broader interest; yet, the editors, in their rebuttal, state that they are “not persuaded” that the evidence presented is “substantial enough to justify publication in [Journal 3] rather than a more specialized journal.” Referee 2 from [journal 5] says that their discovery “that the gut microbiota could influence/canalize the development despite genetic variation is interesting and of importance beyond the field of *Drosophila*”, but states that the manuscript is not of “sufficient general interest” for publication; Referee 3 from the same journal enthusiastically claims that “this is a striking new result, of some interest to a broad range of biologists (not only evolutionary geneticists)”, writing later in her comments that “it should have a substantial impact in the field of microbiota studies”.

Peers find the IPS Lab’s results difficult to grapple with. But beyond such an interpretational controversy, there is an epistemological question about what the evidence of the buffering effect represents. Arbitrating this question of representation and representational scope is the bulk of the reviewers-authors exchanges: here, we develop this theme by relying on Ankeny and Leonelli’s analysis of the characteristics of model organisms (Ankeny & Leonelli, 2011)—of which *Drosophila* is a classic example. We take the freedom of matching their analytical treatment of organisms’ representativeness with an assessment of the representativeness of the buffering story. It is useful to specify here, for the understanding of what follows, that Ankeny and Leonelli distinguish model organisms as a special category of experimental organisms. They are qualified as such because of their (a) material (high degree of standardization, genetic tractability, etc.), and (b) social specificities (communities of users are highly structured, share a common ethos, infrastructures, stock centers, cyberinfrastructures, databases, ways of making knowledge, etc.). Of note, the reviewers’ concerns with the buffering story touch upon this second characteristic of model organisms: in fact, the IPS Lab makes an unscripted use of the lab’s experimental system (cf. Akrich, 1997) and such a use is surprising for their peers. Yet, of importance is also the epistemic trait of distinction that Ankeny and Leonelli attribute to model organisms, which consists (c) of their very broad representational scope. To matter as tools of knowledge-making, model organisms, must be “representative” of biological processes—in genetic/mechanistic or physiological/systemic terms—that are broadly shared by higher organisms, especially human beings.

The negotiations around the representational scope of the buffering story reveal therefore the by-product of an unscripted use of these experimental systems in the IPS Lab: the epistemic orientations (e.g. uses, traditions, ways of knowing) embedded in a given model matter in the selection of such models and therefore also in making the success of the story one tells with it. Complementing the insight of existing scholarship on the social and epistemic considerations that go into the selection of a model organism (cf. Dietrich et al., 2020), our findings offer insight into what happens when expectations about the uses of a model organism (i.e., *Drosophila*) in a given community (i.e., mechanistic biological research) are broken in experimentation. As it will be clear below, the consistency and creativity of unscripted uses dictate the debacle and failure of models in producing valuable knowledge.

In what follows, we document this claim. Specifically, we illustrate how: (1) the use of a genetically diverse heterozygous fly population (i.e. breeding the DGRP pure lines) undermines the possibility for Hal and colleagues to coherently produce the mechanistic claims (based on genetic variation) typical of *Drosophila* research; (2) the tweaking of the experimental systems of the lab raises a debate about the artefactual nature of the buffering story; i.e., it questions whether this evidence speaks as evidence of symbiosis and development to ‘fly people’ understood as relevant community of practice (cf. Craver & Dan-Cohen, 2021).

### Tinkering with the tools means tinkering with the script

As shown above, “in the initial stage of the project (…) [IPS Lab members] were focused on finding candidate genes rather than looking at phenotypic variations”. Failing to “find” the host’s genetic variations “responsible” for the Symbiosium-Growth Effect the group decides to retune its tools, ‘woman-power’ and data and to reinvest them toward another seemingly more productive question. As stated above, a study oriented towards decomposing the genetic and mechanistic components of the Symbiosium-Growth Effect becomes a phenotypic- and variance-oriented one. This realignment of the lab’s experimental systems towards the documentation of the buffering phenomenon implies making unscripted uses of the gene-oriented and genetically controlled DGRP tool. Making it the right tool for the new job entails diverting its use and configuration in one main way: the cross-mating of the lines to replace the genetic homogeneity of homozygous DGRP lines with a genetically heterogeneous population. The whole strategy of diverting DGRP’s use is clearly explained in what the team answers to a reviewer:

‘We thought that these [crossed] strains represent well the idea that we aimed to put forward in the manuscript: that the larvae grow very differently as axenic [i.e germ-free] ones, but very similarly as mono-associated ones. The parental lines are inbred homozygotes [...]; creating F2s shuffles the genetic variants around [...]. Therefore, whatever phenotype is observed in F2 should not necessarily be expected in the homozygous parental lines.’ (Team’s answer to a referee)

The divergence in the use of the DGRP is of great importance. While qualified as a “nice and novel use of the DGRP resource” by a reviewer, it is nonetheless a pivotal element in the editorial resistances we are studying. As Hal explains in the following excerpt, crossing the DGRP meant moving away from the kind of knowledge the DGRP is intended to make. The scripted use of DGRP—which consists of neatly dissecting genotype-to-phenotype maps in a controlled, homozygous population—gets simply lost in the lab’s tweaking of this tool.

We created a population where the genome was variable from individual to individual in a way that wasn't really controlled […] As a result, the gene and genotype can no longer be the object of study, […] I think that if we had succeeded in identifying the exact genotype for each of the individuals […] perhaps that would have interested them... yes, because we would have been back to questioning the genotype to phenotype link. (Interview Hal)

### One does not simply change the geography of causes

Thus, by producing a genetically diverse, non-standardized population, Hal and his group inescapably deviate from the molecular mechanistic program they are expected to advance. While the rationale to do so is the very point they consider groundbreaking in their work—i.e., *Symbiosium* homogenizes the flies’ phenotype *regardless* of the flies’ genotype—this choice is not only surprising for the community, but also jeopardizes the potentialities of their experimental systems. Indeed, their tools turn ineffective within the program established by the Drosophilists for the DGRP and stop offering a viable outlook that can legitimately address the puzzle of symbiosis. The reason lies in the mechanistic explanation they are meant to offer against this phenomenon. As we mentioned above, this would require decomposing the buffering phenomenon into its host-related and microbe-related components, possibly intervening, through manipulations, into switching these components on and off to probe their orchestrated effect on the phenotype (Bechtel & Abrahamsen, 2005; Bechtel, 2013). But with the lab’s decision to tweak their standardized lines, Hal laconically admits, the same experimental system “that allowed them to demonstrate this phenotype does not permit to interrogate the buffering mechanism.” Because of the “complex genetic architecture [that] contribut[es] to the [buffering] phenotype” (team’s answer to a referee), the “classic genetics” they are required to provide to make this demonstration simply “cannot be easily done” in terms of time, ‘woman-power’, money and collaborative possibilities. In a nutshell, pushing the centrality of genetics in the background of their study (and of the tools they employ), the team aims to demonstrate that the genetic background of the flies is *not* the explanans of the phenomenon they study. And yet, their experimental practice falls short of demonstrating the positive claim that the phenotype is a function of development, symbiosis, interaction, and environment.

In the analytical terms of the present work, Hal and colleagues fail to produce the scripted mechanistic molecular explanation baked into the tools they employ (cf. Akrich, 1997), and that is required for publishing these observations in major biology journals for so-called Drosophilists. These tools offer a familiar geography of causes to Hal and other Drosophilists’, they produce explanations that are central to molecular geneticists’ customary programs. Thus, the editorial dead-end observed in our case study is a vibrant example of the difficulty to change these explanations (through the tweaking of the tools that perpetuate them). “How gut symbionts promote phenotypic buffering remains unclear”, says editor 1 to justify the editorial rejection (i.e., rejecting the paper without sending it to reviewers). Unclear, one could add through the voice of Editor 4, because of the “dearth of experimental mechanism” in the story presented by the IPS Lab: simply put, among Drosophilists “a phenotype is not enough” (Hal interview).

### Representing organisms or artefacts?

Not only does the drawing of an unscripted geography of causes turn the tweaked tools ineffective with respect to the script that structured these tools epistemically and socially. But, this tweaking results in the tools getting questioned as such. What are these experimental systems worth under their unscripted use? Issues around the epistemic value of the study take on its representational scope and specifically can be characterized as an ‘artefactuality critique’:

“I had a large problem with the fact that the study is based on the comparison of the response of organisms either in a totally artefactual stressful condition, in which no organism ever evolved (axenic), or in a mono-association (with a bacteria). Furthermore, I don't really understand why the experiments were performed under chronic under-nutrition conditions, if we don't know whether adult flies face this condition in nature. One can easily think that *Drosophila* larvae do not live naturally in poor environments because females choose a site that is generally rich in rotten fruits. Of course, larval competition could trigger resource limitation but there is to my knowledge no work (or even observation) suggesting that.” (Referee 2, Journal 5; emphasis added)

Thus, as the experimental system is being stretched toward the investigation of an unscripted representational target, its very ability to produce evidence for something else than itself becomes a matter of doubt and negotiations. Hereabove, we see one example of how peer reviewers and editors call into question how much of ‘nature’ the *Drosophila-Symbiosium* model takes up. While artefactuality can be seen as the very bedrock of the experimental styles of reasoning of laboratory sciences (Hacking, 1992; Rheinberger, 1997a), as well as a constitutive element of model organisms (Ankeny & Leonelli, 2021), it is here considered a major limitation to the representational power of the team’s evidence.^7^ This provides a major argument for the rejection of the paper. A straightforward formulation of this criticism comes from referee 2 and an additional expert summoned into the agon after the team appealed against the editorial decision of rejection in [Journal 5]. Their comments mostly insist on the *artefactual*—and consequently irrelevant—character of the study. In advocating for a definitive rebuttal, the additional expert argues: “The absolute unnatural conditions of growing without bacteria” (Referee 2) point towards the experimental irrelevance of the paper because “nature never sees a germ-free fly” (Expert). The “study's real-world implications are lacking” (Expert), because the paper is based, according to this expert, on “totally artefactual stressful condition” that the authors have *made up* to explore the complexity of multispecies integrations in developing flies. If the team’s claim—the reviewer and expert argue—is an affirmation of the effects of environmental stressors on the canalization program of the fly’s genome, then the results are not new and rather uninteresting to the community. If, instead, the study sets out to demonstrate the functioning of complex multispecies symbioses in normal development within stressful conditions, then their experimental system is not proper ground to produce evidence for this claim.

To this critique of artefactuality, the team answers with a different interpretation of the experimental system they have devised, and the type of questions it allows them to explore. Thanks to the combination of environmental stress (i.e. undernutrition) and the condition of axenicity, they can excavate a different role for Symbiosium in the “canalization program” of the fly’s development. In their view, they may be lacking a mechanism, but they hold a clear experimental target and a rather innovative one: the fly’s developmental programs (the target) rest on a molecular machinery that reaches outside the host’s cells and genome—that is, into the organism’s symbionts. While referee 2 and the expert take the axenic fly to be only an artefact—the *degré zéro* of representativeness—the team argues for the axenic fly as setting the stage for causally inferring how partners in symbiosis (which they can manipulate experimentally) are not merely a host and a guest. Rather, they thoroughly shape one another in a unique trajectory of development that they both produce and concretize in an organism.

Not only the conception of what it has to be done in a biological laboratory is at stake in these exchanges, but the ‘artefactuality critique’ also deeply questions how much of unscripted tweaking a tool can take before it loses its epistemic value. Or, put another way, the actions of the IPS Lab team suggest they needed *other tools*—maybe the “whole suite of postgenomic techniques” Medina and Sachs were writing about (Medina & Sachs, 2010)—to answer the questions they were asking. More than the requirement to make “more experiments”—as hoped for in interviewing by Hal and Hui—the disagreement is conceptual: it is about the indicative relationship between the target phenomenon (i.e. the *Symbiosium* Growth Effect) and the explanations afforded by an arrangement of experimental systems. The tools and organisms at disposal in the IPS Lab are taken and tweaked to illustrate an under-scrutinized phenomenon—and more, a phenomenon that these tools are not scripted to investigate in the first place. Thus, the team acknowledges the fallibility and limitations of their experimental configuration: “we are demonstrating what is biologically feasible in a defined set of conditions” they write. As Hal candidly admits in an interview, they are well aware that “in an extremely factual and sincere way [their] model represents only itself”. But the *biologically feasible* has, in and of itself, limited epistemic value for the reviewers: the set of conditions that the rearranged tools define do not allows the team to disentangle mechanistically the components of symbiosis, and to affirm the relevance of these processes as they are expected to—that is, by establishing their causal contribution. The critics of the IPS Lab’s paper, and particularly the ‘artefactuality critique’, thus emphasize how the IPS Lab’s study violates the “norms implicit and explicit in the practice of designing, executing, and interpreting experiments” (Craver & Dan-Cohen, 2021). This, in fact, is what makes the *Drosophila-Symbiosium* model an artefact no longer intelligible to other Drosophilists who study symbiosis.

By being stretched too far beyond its scripted reach, the *Drosophila-Symbiosium* model is socially and epistemically destabilized in such a way that it loses its status as knowledge-maker. The question then arises of how much of epistemic freedom a tool allows; can we draw new causal geographies with tools in and through which ‘old geographies’ are scripted, or do new geographies require new tools? As Hal explained in an interview, the team “wanted to tackle *concepts* and manipulate them with [their] experimental systems” (emphasis added). But while ‘epistemic surprise’ is the very essence of experimental systems (Rheinberger, 2020), the question is then the possibility a given experimental system offers to stabilize unscripted concepts into new epistemic objects.

## Discussion

In the beginning, interrogating the IPS Lab’s experimental systems meant enacting a specific social and technical script (Akrich, 1997). This script concerns the kind of questions these experimental systems can and are expected to answer; specifically, we argued, questions about *the fly’s genetic traits associated with—and therefore causative of—the Symbiosium Growth Effect*. The script also entails a geography of causes explaining this phenomenon: a specific type of mechanistic explanation has to be produced to make sense of the symbiosis’ canalization effect. This explanation emphasizes the role of the genetic components of host–microbe interaction in orchestrating specific phenotypical outcomes (cf. Bechtel & Abrahamsen, 2005; Bechtel, 2013). Yet, this experimental system, like many (Rheinberger, 2011), behaves unexpectedly: in the IPS Lab, genotypic variables *failed to explain* phenotypic differences in gnotobiotic DGRP flies. In the face of this observation, the IPS Lab is forced to tweak its experimental systems and tools in search of an explanation that fits their observation (or lack thereof). Thus, these scientists shift their use of the DGRP tool from an instrument to address the role of the nuclear genome in phenotypic development, to a study of the role of symbiosis in explaining the fly’s developmental variability (e.g. growth and length). This new story realigns the lab’s work behind a different explanation of host–microbe interaction: one based on symbiosis and host–microbe interactions more than on the genetic control of this process.

However, these unscripted uses of the lab’s experimental systems are met with skepticism by the reviewers of the paper reporting this evidence. Specifically, the lab’s explanation of the *Symbiosium* buffering effect gets contested on mixed social and epistemological grounds. As argued by Ankeny and Leonelli (2011), an experimental organism has both representational target and representational scope. While the representational scope consists of “how extensively the results of research with a particular experimental organism can be projected onto a wider group of organisms”, the representational target is defined as “the phenomena to be explored through the use of the experimental organism.” In the IPS Lab, the team's effort to switch to a different explanation of symbiotic development equals a change in the representational target of their experiments. They move from the question of *finding the mechanism* for the Growth Effect to *showing the canalization* role of Symbiosium in *Drosophila* development. This change in target of their experiments is not devoid of consequences. As we detailed above, their move is a deviation from customary uses of their tools: these no longer fit with the uses inscribed into their design and circulation in the Drosophilist community. This practice of craft and inventiveness jeopardizes the epistemic value of these experiments. The result is the destabilization of the representational scope of the IPS Lab’s study—if not its dramatic demise. Tweaking the script of the lab’s tools calls into question whether the lab's experimental systems and data are still adequate to tell something meaningful about their target. New questions emerge: about experimental errors, or about artifactuality of the *Drosophila-Symbiosium* model, or about the legibility this model offers of naturally occurring phenomena (i.e., external validity). The controversy around the representational value of the buffering story reveals, in analytical terms, that unscripted uses of life sciences’ experimental systems can easily slip into unacceptable deviations: from bedrocks of developmental genetics, an unconventional use of DGRP can turn the *Drosophila* model into suspicious artifact. Our findings add therefore to the recognition that social and epistemic expectations mix in the choice of a research organism (cf. Dietrich et al., 2020). They illustrate how these criteria get traded off in practice; thus, providing an empirical depth to the claim that specific epistemic advantages of one choice (e.g., tweaking the tools to enhance novelty of research) can come at the cost of devaluing other features of the research organism (e.g., its representational scope). More importantly, our paper demonstrates how these trade-offs are far from finding univocal resolution and are contingent to a situated setting: what makes *Drosophila-Symbiosium* a model organism in the community of mechanistic biological research depends also on editorial choices that have resolved this controversy, or the vicissitudes of peer reviewing. Heterogeneous factors play a role in defining the limits of the model’s unscripted uses in ways that are meant to avoid the model’s debacle and sanction unacceptable uses in negotiation.

Finally, our article inquiries into the present postgenomic moment in the life sciences, and specifically the so-called ‘microbiome revolution’. More specifically, we offer here a much-needed empirical account of the technoscientific settings (e.g. research designs, tools, ideas, relations, collaborations, etc.) that make up the so-called microbial turn. Is this microbiome research, as exemplary postgenomic practice, a distinct research program from genomics or classical genetics? The case of the IPS Lab can help us provide a nuanced answer to this question and offer an empirical documentation of how scientists enact these unresolved tensions in postgenomics. Reframing the organism as a multiplicity of reactive genomes (cf. Dupré, 2010) means less producing “radical novelty” (Morange, 2018, p. 189) than it means producing a story that just deviates from the one scripted into the sociotechnical experimental systems of post-HGP life sciences. Leaving aside rhetorical talks of the postgenomic “microbiome revolution” (Blaser, 2014), the coherent enactment of this research agenda entails experimental digressions that *both* build upon *and* challenge the established ways of scientists for procuring, selecting and producing evidence in the post-HGP era. Our article complements therefore views of ‘postgenomics’ as novel theory and representation of biological phenomena (cf. Fox Keller, 2015; Dupré, 2010), or as the rebranding of the old genomics for sustained funding (Morange, 2018). Our work rather offers a *pragmatic understanding* of the so-called microbial turn in postgenomics. On the one hand, this moment in the history of the life sciences is, if anything, a set of daily social, technical and epistemic challenges for scientists. Rearrangements and recombinations of the lab’s experimental systems (Rheinberger, 2011, 1997b) are needed in order to produce this knowledge. Far from being uncontroversial, these adjustments have to be stabilized and can be the subject of dispute and negotiation among peers in the context of publication. On the other hand, the microbial turn is a pragmatic endeavour also in the sense of having little to do with a change in the fundamental ontological foundations of biology (cf. O’Malley & Dupré, 2005). At stake in the IPS Lab is not settling the question as to whether the *Symbiosium-Drosophila* enacts (or not) a paradigm-shift into a novel ontology of the holobiont. Rather, our article shows a practical preoccupation of these scientists: their goal is to turn the established ways of knowledge-making, tools and epistemic practices of today’s molecular biology labs into the technoscientific infrastructure to apprehend the body as a plastic assembly of human and microbial cells and genomes. This entails, for the actors involved, producing coherent factual claims from their configuration of experimental systems; negotiating it with others; trading off the feasibility, convenience and novelty of their practices. Notably, this process entails also taking considerable risks while also being a productive endeavor that opens novel representational spaces for the studied phenomenon (i.e. developmental symbiosis). Without the need to deny that the life sciences are “becoming increasingly ambitious”, and that the “trend now” is towards an integrated view of the organism (Guttinger & Dupré, 2016, p. 31), such a pragmatic approach to postgenomics permits therefore to delimit the relations of justifications, evidence, tools, research designs, and trust that make up so-called postgenomic representations of life. A messy, heterogeneous gamut of technoscientific practices may be the defining feature of postgenomics. This certainly requires further historical, philosophical, and sociological scrutiny in the years to come.

## Footnotes

1 The word “microbiota” refers to a community of microbes living in a given environment (be it a host or a gut, be it at sea or in soil). The word “microbiome” refers to the sum of the bacterial genomes of a microbiota, that is to the result of metagenomic analysis of the bacterial community in a given environment. In this sense, "the microbiome revolution" can be understood here as a socio-epistemic phenomenon producing the conditions of possibility of a thriving microbiome science (i.e. development and diffusion of metagenomics).

2 Institutional and individuals’ names are fictitious for the purpose of de-identification.

3 A computational procedure drawing statistical association between complex traits (i.e. traits that exhibit a continuous range of variation and are influenced by both environmental and genetic factors) and specific genetic variations.

4 As described in the supplementary material: “All live *Drosophila *larvae were collected from each nutritive cap containing low yeast diet by temporary immersion in sterile PBS, transferred on a microscopy slide, killed with a short pulse of heat (5 s at 90 °C), mounted with 80% glycerol/PBS. The images were taken with the stereomicroscope and the lengths of individual larvae were measured using ImageJ software. For each DGRP strain and each cross and/or condition, at least three biological replicates were generated.

5 On model’s ability to « surprise» see (Rheinberger, 1997b; Keller & Winship, 2002; Morgan, 2005).

6 The concept of canalization/buffering refers to the ways developmental processes lead to similar phenotypes in a population despite genetic and environmental variations. “The constancy of the wild type must be taken as evidence of the buffering of the genotype against minor variations not only in the environment in which the animals developed but also in its genetic make-up.” (Waddington, 1942, p. 564).

7 This is why, following an emerging trend of studies, we employ the language of “artefactuality” and “artefact” to speak about the critique of the *Drosophila-Symbiosium* story. See (Craver & Dan-Cohen, 2021) for a philosophical treatment of experimental artefacts and the developing literature on this matter.
